# Effectiveness of Omega-3 Supplementation on Arterial Stiffness: A Systematic Review

**DOI:** 10.3390/medicina62081553

**Published:** 2026-08-12

**Authors:** Jacqueline Marlene Ibarra-Peso, Mariel Alejandra Lobos-Farias, Felipe Antonio Albarrán-Torres, Erislandis López-Galán, Miguel Enrique Sánchez-Hechavarria

**Affiliations:** 1 Departamento de Ciencias Clínicas y Preclínicas, Facultad de Medicina, Universidad Católica de la Santísima Concepción, Concepcion 4090541, Chile; jibarra@ucsc.cl (J.M.I.-P.); mlobos@ucsc.cl (M.A.L.-F.); 2 Departamento de Ciencias Básicas y Morfologia, Facultad de Medicina, Universidad Católica de la Santísima Concepción, Concepcion 4090541, Chile; falbarran@ucsc.cl; 3 Departamento de Ciencias Básicas Biomédicas, Facultad de Medicina 2, Universidad de Ciencias Médicas de Santiago de Cuba, Santiago de Cuba 90100, Cuba; erislandisl@gmail.com; 4 Núcleo Científico de Ciencias de la Salud, Facultad de Ciencias de la Salud, Universidad Adventista de Chile, Chillan 3780000, Chile

**Keywords:** arterial stiffness, pulse wave velocity, omega-3 fatty acids

## Abstract

*Background/Objectives***:** Evidence linking omega-3 intake to reductions in arterial stiffness remains inconsistent. This systematic review aimed to evaluate and synthesize evidence on the effectiveness of omega-3 supplementation in reducing arterial stiffness as measured by pulse wave velocity (PWV). *Methods*: Two search iterations were conducted in Scopus, PubMed, and Web of Science. The search was conducted through 2025. The search terms were “omega-3” AND “pulse wave velocity”. No filters for language or study type were applied. *Results*: In this set of 20 randomized controlled clinical trials, omega-3 supplementation had heterogeneous effects on arterial stiffness measured by PWV. Slightly more than half of the studies reported significant reductions in PWV, while the remaining studies reported no statistically significant changes. This heterogeneity may be explained by differences in study population, dose, and treatment duration. Favorable effects were more commonly observed in studies using moderately high omega-3 doses (approximately 1.8–4 g/day), interventions lasting ≥12 weeks, and populations with metabolic disorders or higher vascular risk. In contrast, studies using low doses, acute interventions, or plant-based omega-3 more frequently reported null results. *Conclusions*: These findings indicate that population characteristics, dose, and intervention duration should be considered when designing future trials and clinical recommendations.

## 1. Introduction

Cardiovascular diseases represent the leading cause of death worldwide. By 2030, annual mortality from cardiovascular diseases is projected to exceed 23.6 million deaths [1]. Multiple cardiovascular risk factors are prevalent in the general population [2], and cardiovascular risk assessment guides pharmacological treatment and lifestyle interventions, including dietary modification and physical activity [3]. Atherosclerotic disease, prevalent even in young adults, progresses silently from childhood, increasing arterial stiffness through lipid accumulation, inflammation, and vascular remodeling [1,4]. Aging and factors such as hypertension further accelerate the loss of arterial elasticity [4], making arterial stiffness a key marker of cardiovascular risk [5].

Among arterial stiffness indices, carotid–femoral pulse wave velocity (cfPWV) has gained prominence and is considered the “gold standard” [6]. Pulse wave velocity (PWV) is determined by measuring the transit time between two arterial sites and the distance between them [7]. Age, environmental factors, inflammatory mediators, fatty acid-binding proteins, and genetic factors (polymorphic variants of fibrillin-1, angiotensin II type 1 receptors, and endothelin receptors) contribute to the multifactorial nature of structural and functional changes in the arterial wall [8,9]. Interventions such as exercise, dietary modification [10], lipid control, anti-inflammatory measures [11], and microbiota modulation [12] have demonstrated effects on arterial stiffness measured by PWV in various clinical contexts.

It has been suggested that dietary composition modulates arterial stiffness, as reflected by changes in PWV [13,14,15]. Individuals with a higher saturated fatty acid intake exhibited the highest rates of arterial stiffness progression [16]. However, overweight is not always associated with greater arterial stiffness than in normal-weight individuals, and stiffness is independently associated with increases in arachidonic acid/linoleic acid (AA/LA) ratios and decreases in eicosapentaenoic acid/arachidonic acid (EPA/AA) ratios [17]. Conversely, high intake of EPA and docosahexaenoic acid (DHA) may help protect non-carriers but not carriers of the high cardiovascular risk allele [18]. Although isolated evidence suggests that higher consumption of oily fish late in pregnancy is associated with lower infant aortic stiffness [19], and that EPA intake improves arterial stiffness by reducing inflammation in obese patients with dyslipidemia [20], the evidence from randomized controlled trials (RCTs) remains inconsistent and inconclusive. Some RCTs report significant reductions in PWV following omega-3 supplementation, particularly in populations with elevated baseline cardiovascular risk or inflammation. However, others show null or even opposing effects, possibly due to differences in dosage (e.g., EPA vs. DHA proportions), intervention duration, baseline omega-3 status, or the heterogeneity of the populations studied (e.g., healthy vs. diseased, normotensive vs. hypertensive). Furthermore, variations in PWV measurement protocols and the use of different arterial stiffness indices across studies complicate direct comparisons. Consequently, a critical gap persists: it remains unclear under which specific conditions (e.g., population, dose, duration, and baseline omega-3 status) an omega-3-rich diet or supplementation reliably reduces arterial stiffness as assessed by PWV. This lack of synthesis and mechanistic understanding hinders the translation of omega-3 interventions into clinical guidelines for vascular health. Therefore, a systematic evaluation of the existing RCT evidence is warranted to clarify the effect of omega-3 on PWV and to identify the factors contributing to the observed heterogeneity in outcomes.

## 2. Materials and Methods

**Figure 1:**
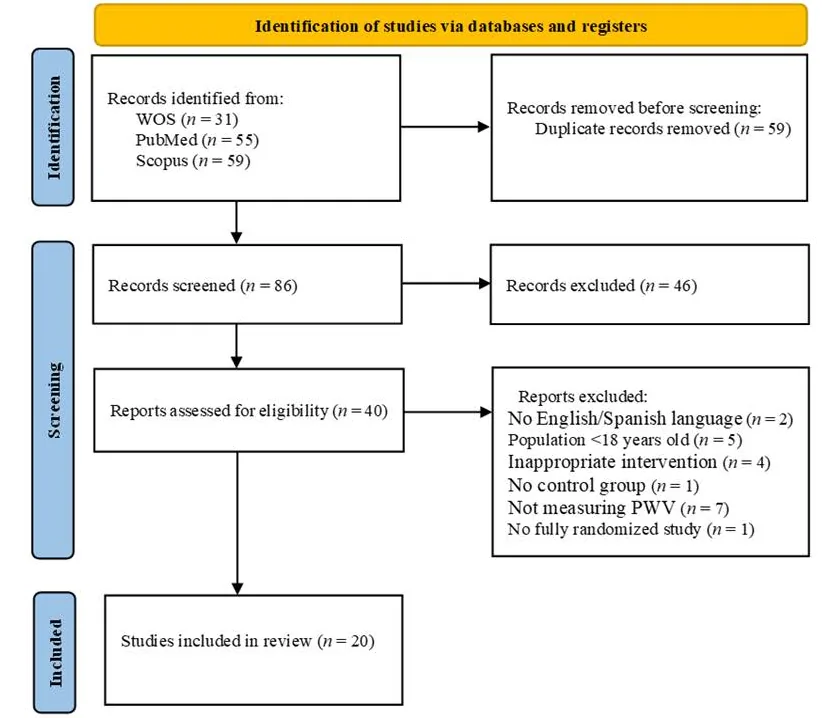
Flow chart depicting the selection process of the studies included in the review.

This systematic review was conducted and reported in accordance with the Preferred Reporting Items for Systematic Reviews and Meta-Analyses (PRISMA) 2020 statement [21]. The completed PRISMA 2020 checklist is provided as Supplementary Table S1, and the study selection process is summarized in the PRISMA 2020 flow diagram (Figure 1).

A search and compilation of scientific documents were conducted to analyze publications on omega-3 supplementation and its effect on vascular stiffness measured by PWV.

Research question: What is the effect of omega-3 on vascular stiffness measured by PWV in adult humans with or without cardiovascular comorbidity?
Eligibility criteria (PICOT):
Patients: Adult humans with or without cardiovascular comorbidity, non-pregnant.
Intervention: Omega-3 supplementation, defined as any pharmacological or dietary supplementation rich in omega-3, clearly specified by the primary study authors.
Comparison: No omega-3 supplementation or other treatment.
Outcome: Arterial stiffness, defined as PWV.
Study type: Randomized clinical and cross-over trials.
Search strategy

Two iterations were conducted in the Scopus, PubMed, and Web of Science (WoS) databases, with the final search performed on 1 December 2025. No filters for language or study type were applied to the search. Duplicates were removed during this phase using the Zotero software (version 7.0). The keywords used for search and classification were: “omega-3” AND “pulse wave velocity.” Two reviewers (MESH and ELG) independently screened the titles and abstracts for articles and read the full text for the potentially eligible studies. Conflicts were discussed and resolved by the third, fourth, and fifth senior reviewers (FAAT, JMIP, MALF). The following studies were excluded: duplicate publications, reviews, editorials, case series, case reports, guidelines, basic research, preprint papers, studies that do not provide PWV data, and studies that include children as subjects. The selection process is shown with a flow chart (Figure 1).

Data analysis and extraction

The selected articles were classified according to general variables such as: title, authors, year, abstract, keywords, database, article accessibility, article type, study type, sample, and the Cochrane Risk of Bias 2 (RoB 2) [22], which allows for the assessment of the quality and internal validity of the selected articles.

## 3. Results

The number of records retrieved from the searches conducted in the different databases was as follows: WOS (*n* = 31), PubMed (*n* = 55), and Scopus (*n* = 59), totaling 145 records. After the removal of duplicates (*n* = 59), a total of 86 records were subjected to the initial screening phase, during which they were assessed based on study type. In this stage, 46 studies were excluded: 14 were reviews; 1 was a design and rationale study; 1 was a letter to the editor; 1 was a book chapter; 1 was an expert panel consensus; 3 were animal studies; 23 were observational studies; and 2 were theoretical models. Following this screening by study type, 40 records were retained, of which 2 were excluded because they were written in Russian, resulting in 38 records that were analyzed in detail. In this phase, 17 studies were excluded: 5 for including participants under 18 years of age; 4 for involving an inappropriate intervention; 1 for lacking a control group; and 7 for not measuring PWV. Ultimately, 20 articles satisfied all eligibility criteria and the objectives of this review (Figure 1).

Table 1 presents the variables related to the study’s thematic focus. These include the characteristics of the study subjects and the instruments used to measure vascular stiffness. The parameter employed to assess arterial stiffness is also specified, along with the structure and composition of the dietary intervention. Additionally, the table outlines both the explicit and implicit limitations of the studies.

Finally, the findings related to the objectives of this literature review are presented, accompanied by the risk-of-bias assessments obtained using the Cochrane RoB 2 tool [22].

**Table 1:** Overview of the studies included in the systematic review.

N	Article	Subjects	Intervention	Measured Outcomes	Instruments	Limitations	Results of PWV	RoB2
1	[18] AlSaleh A et al. (2014)	310 healthy adults. Age 45–70 years.	Placebo capsules or EPA and DHA (in a 1.51:1 ratio) in three different doses (0.45, 0.9 and 1.8 g/day) at regular intervals for 12 months.	cfPWV.	SphygmoCor.	Only one genetic variant analyzed.	No significant effect on cfPWV were observed when comparing doses of 0.45, 0.9 and 1.8 g/day of EPA and DHA with placebo, regardless of the genotype of the single nucleotide polymorphism (SNP) rs1,378,942; located in intron 1 of the c–Src (CSK) tyrosine kinase gene.	Low risk
2	[23] Satoh N. et al. (2009)	92 patients with obesity, dyslipidemia and metabolic syndrome. Age 51.7 ± 1.5 years.	Diet + 1.8 g/day of pure EPA) or diet alone. Duration 12 weeks (3 months).	baPWV.	Vasera VS 1000 (Fukuda Denshi, Tokyo, Japan).	No double blinding.	Significant effect of omega-3 on baPWV (*p* < 0.01).Omega-3group: -Before: 14.00 ± 0.39 m/s. -After: 13.21 ± 0.32 m/s. Control group: -Before: 13.88 ± 0.40 m/s. -After: 13.99 ± 0.41 m/s.	Some concerns
3	[24] Takaki A. et al. (2011)	50 patients with dyslipidemia and coronary artery disease. Age 61.6 ± 5.6 years.	Daily dose of 1.8 g EPA for 48 weeks (12 months). Duration: 12 weeks (3 months).	baPWV.	AT–form VOP/ABI (Omron Colin, Tokyo, Japan).	The duration of pharmacological treatment and the level of adherence were not monitored. Dietary patterns were not monitored.	There were no significant effect on baPWV between subjects who had statin–only treatment 16.70 (15.85–17.93) m/s and those who had combination therapy with EPA 15.52 (13.85–16.84) m/s, although the increase in the mean baPWV value from the baseline value in the statin–only group (0.909 m/s) was greater than in the combination therapy group (0.441 m/s).	Some concerns
4	[25] Siasos G. et al. (2013)	20 healthy smokers. Age 27.63 ± 2.65 years.	Treatment group: Oral supplementation with omega-3 fatty acids (PUFAs), at a dose of 2 g/day (46% EPA + 38% DHA). Control group: Placebo. Duration: 12 weeks (3 months).	Carotid–femoral pulse wave velocity (cfPWV).	SphygmoCor.	Small sample size.	Significant effects of omega-3 on cfPWV (*p* = 0.007). Omega-3 group: -Day 0: 5.87 ± 0.63 m/s. -Day 28: 5.84 ± 0.64 m/s. -Day 84: 5.54 ± 0.76 m/s. Control group: -Day 0: 5.87 ± 0.64 m/s. -Day 28: 5.84 ± 0.69 m/s. -Day 84: 5.74 ± 0.58 m/s.	Low risk
5	[26] Tousoulis D. et al. (2014)	29 patients with metabolic syndrome. Age 44 ± 12 years.	Omega-3 (2 g, 46% EPA and 38% DHA) or placebo, for 12 weeks (3 months).	cfPWV.	SphygmoCor.	There is no control of dietary patterns. Small sample size.	Significant effect of omega-3 on cfPWV (*p* < 0.001). Omega-3 group: -Before: 7.62 ± 1.59 m/s. -Day 28: 7.40 ± 1.84 m/s. -Day 84: 7.22 ± 1.54 m/s. Control group: -Before: 7.58 ± 1.77 m/s. -Day 28: 7.58 ± 1.76 m/s. -Day 84: 7.54 ± 1.72 m/s. N rs	Low risk
6	[27] Fukuoka Y et al. (2014)	Patients with low cardiovascular risk (*n* = 106), high cardiovascular risk (*n* = 23), and coronary artery disease (*n* = 62). Age 70.3 ± 10.4 years.	Fish–rich diet vs. EPA supplements (1.8 g/day) for 6 months.	baPWV.	Blood pressure and pulse wave device (BP–203RPE II form VOP/ABI; Omron Healthcare Co., Ltd, Tokyo, Japan).	Without a pure placebo group.	Significant effect of fish diet on baPWV in the low–risk group after 6 months (*p* = 0.0315). -Before: 18.07 ± 0.370 m/s. -After: 17.73 ± 0.344 m/s. No significant effect of fish diet on baPWV in either the high cardiovascular risk group (*p* = 0.7350). -Before: 18.96 ± 0.369 m/s -After: 18.82 ± 0.404 m/s No significant effect of fish diet on baPWV in the group with coronary artery disease (*p* = 0.0686). -Before: 18.25 ± 0.351 m/s. -After: 18.64 ± 0.349 m/s. Significant effect of fish diet on baPWV in 21 patients with coronary artery disease and high risk assigned to sequential treatment with pure EPA (*p* = 0.0061). -Before: 19.68 ± 0.344 m/s. -After: 18.29 ± 0.344 m/s.	High risk
7	[28] Casanova MA. et al. (2017)	29 hypertensive patients with low and high cardiovascular risk and hypertriglyceridemia. Age 40–65 years.	1.8 g/day (60% EPA and 40% DHA) or ciprofibrate 100 mg/day for 12 weeks (3 months).	cfPWV.	Complior.	Dietary patterns were not monitored before and after the intervention; there is no comparison between baseline and baseline during the washout period, potentially resulting in a residual effect; there is no control group. Small sample size.	High cardiovascular risk group: significant effect of omega-3 on cfPWV (*p* = 0.021). -Before: 10.4 ± 0.4 m/s. -After: 9.4 ± 0.3 m/s. Low cardiovascular risk group: no significant effect of omega-3 on cfPWV (*p* = 0.305). -Before: 9.0 ± 0.5 m/s. -After: 8.7 ± 0.5 m/s.	High risk
8	[29] Bunout D. et al. (2021)	100 patients with chronic kidney disease in the pre–dialysis phase. Age 56–74 years.	Omega-3 group: 4 g/day of fish oil, providing 3.666 g/day of DHA and EPA. Control group: Corn oil. Duration: 12 weeks (3 months).	PWV.	Complior.	Corn oil is not an inert placebo. High variability in PWV measurements.	Significant effect of omega-3 on PWV (*p* < 0.05). Omega-3 group: -Before: 9.75 (8.1–10.6) m/s. -After (week 6): 10 (7.6–10.8) m/s. -After (week 12): 10.1 (8–10.9) m/s. Control group: -Before: 9.3 (8.5–10.7) m/s. -After (week 6): 9.7 (8.3–11) m/s. -After (week 12): 9.7 (8.3–11.2) m/s.	Some concerns
9	[30] Meital LT. et al. (2021)	30 men with abdominal aortic aneurysm (AAA), age 74.4 ± 5.3 years compared with a subset of 20 healthy male participants, age 73.2 ± 5.6 years.	Patients received omega-3 capsules (1.8 g; 5:1 ratio of DHA:EPA; *n* = 15) or placebo capsules (49:49:2 ratio of corn oil, olive oil, and fish oil; *n* = 15), for 12 weeks (3 months).	cfPWV.	SphygmoCor.	Limitations of this study include the lack of ultrasound for AAA in participants in the control group. Corn, olive, and fish oils are not inert placebos.	In patients with AAA, a significant negative correlation was observed between cfPWV and the omega-3 index (r = 0.54, *p* = 0.017). Significant effect of omega-3 on cfPWV (*p* = 0.014). Omega-3 group: -Before: 14.2 ± 0.6 m/s. –12 weeks: 12.8 ± 0.7 m/s. Control group: -Before: 14.6 ± 0.6 m/s. -12 weeks: 14.0 ± 0.5 m/s.	Low risk
10	[31] Ando N. et al. (2025)	120 Japanese women. Age 45–65 years.	3 mixtures of edible oils providing 0.97 g/d, 1.36 g/d or 2.13 g/d of ALA, or a placebo providing 0.01 g/d of ALA.	Brachial–ankle pulse wave velocity (baPWV).	HBP–8000 (OMRON Corporation).	ALA was used as a supplement and placebo at a lower dose. Although serum fatty acid concentrations were measured, their metabolites were not analyzed, so it is unclear whether these contribute to the improvement in baPWV.	Significant effect of omega-3 on baPWV (*p* < 0.05). Group 1 (0.97 g/d ALA): -Before: 13.254 ± 1.718 m/s. -After: 12.612 ± 1.475 m/s. Group 2 (1.36 g/d ALA): -Before: 13.586 ± 1.871 m/s. -After: 12.885 ± 1.740 m/s. Group 3 (2.13 g/d ALA): -Before: 13.916 ± 2.297 m/s. -After: 13.127 ± 2.089 m/s. Control group: -Before: 13.415 ± 2.053 m/s. -After: 13.784 ± 2.173 m/s.	Low risk
11	[32] Monahan KD. et al. (2015)	12 young adults (age 21–35 years) and 12 healthy older adults (age 60–80 years).	4 omega-3 capsules (465 mg EPA and 375 mg DHA) a day, in two doses, for 12 weeks (3 months).	PWV of central or peripheral origin.	Two non–directional Doppler flow probes (Model 810 A; Parks Medical Electronics, Inc., Aloha, OR, USA) y (Chart, ADInstruments).	All subjects received the same supplementation, not blinded. Small sample size.	Pulse wave velocity (PWV) was higher in the arm, leg, and aorta of older adults compared to younger adults before omega-3 supplementation (Pre). In younger adults, omega-3 supplementation had no effect on central or peripheral PWV. In contrast, omega-3 supplementation had significant effect on cfPWV (*p* < 0.05) in older adults: -Before 9.88 ± 0.65 m/s -After 8.95 ± 0.65 m/s however, levels after omega-3 supplementation remained higher than in younger adults.	High risk
12	[33] Serhiyenko VA. et al. (2018)	36 patients with type 2 diabetes mellitus and cardiovascular autonomic neuropathy. Age 50–59 years.	1 gram of omega-3 (DHA and EPA) for 3 months.	PWV.	TensioMedTM Arteriograph.	Numerical imbalance of subjects between the groups.	Significant effect of omega-3 on PWV (*p* < 0.01). Omega-3 group: -Before: 11.3 ± 0.48 m/s. -After: 9.0 ± 0.44 m/s. Control group: -Before: 10.5 ± 0.42 m/s. -After: 10.1 ± 0.41 m/s.	High risk
13	[34] Bellien J. et al. (2022)	81 patients with hypertension and metabolic syndrome. Of these, 41 were randomized to receive placebo (age 55.0 ± 7.5 years) and 40 received camelina oil (age 57.7 ± 8.4 years).	Camelina oil 30 ml/day (approximate composition: 30% ALA and 15% omega-6 linoleic acid), for 6 months.	cfPWV o baPWV.	Complior SP, o SphygmoCor.	ALA (vegetable omega-3) may be less potent than EPA/DHA.	No significant effect of camelina oil on cfPWV or baPWV (*p* = 0.70). Camelina oil group: 10.7 ± 2.0 m/s. Control group: 10.4 ± 1.9 m/s.	Low risk
14	[35] Root M. et al. (2013) Control group	Healthy subjects. Treatment group: Age 21.4 ± 2.9 years (*n* = 30). Control group: Age 20.4 ± 2.1 years (*n* = 27).	Specific supplementation: 4 weeks (1 month) 350 mg of EPA and 230 mg of DHA. Placebo: 1.0 g of safflower oil.	baPWV.	SphygmoCor.	Young and healthy population with low baseline PWV (ceiling effect).	No significant effects of omega-3 on baPWV (*p* = 0.26): Omega-3 group: -Before: 7.49 m/s. -After: 7.43 m/s. Control group: -Before: 7.44 m/s. -After: 6.61 m/s.	Some concerns
15	[36] McManus S. et al. (2016)	26 healthy men. Age 36–54 years.	During the three separate study days, at least 4 weeks apart, meals containing EPA–rich oil, DHA–rich oil, or control oil were consumed.	cfPWV.	Vicorder device (Skidmore Medical, Bristol, United Kingdom)	It was not possible to detect 19,20–EpDPE, as it fell below the detection limit; furthermore, the intervention was only performed on men and was short–term. Small sample size.	No significant effect of omega-3 on cfPWV (*p* < 0.05). EPA group: -Before: 8.3 ± 0.1 m/s. -After: 8.4 ± 0.2 m/s. DHA group: -Before: 8.2 ± 0.2 m/s. -After: 8.2 ± 0.2 m/s. Control group: -Before: 8.5 ± 0.2 m/s. -After: 8.3 ± 0.2 m/s.	Low risk
16	[37] Hearon CM. et al. (2022)	56 obese adults at high cardiovascular risk. Age 40–55 years.	Supplementation with omega-3 fatty acid ethyl esters (1.6 g/day) or placebo with olive oil (1.6 g/day) for 12 months.	central PWV (cPWV).	SphygmoCor.	Combined intervention (exercise + Omega-3) makes it difficult to isolate the effect of Omega-3. Olive oil is not an inert placebo.	No significant effect of omega-3 on cPWV (*p* < 0.05): Omega-3 group: -Before: 7.08 ± 0.71 m/s. -After: 7.43 ± 1.70 m/s. Control group: -Before: 7.27 ± 1.60 m/s. -After: 7.15 ± 1.14 m/s.	Some concerns
17	[38] Pipingas A. et al. (2023)	26 healthy men. Age 52.79 ± 5.29 years.	Single dose 4.74 g omega-3 (4020 mg DHA and 720 mg EPA) or placebo (sunflower oil)	cfPWV postprandial.	SphygmoCor.	Results are acute (postprandial); small sample and only men.	No significant effect of postprandial omega-3 on cfPWV (*p* = 0.928). Omega-3 group: -Before: 9.77 ± 0.89 m/s. -After: 9.65 ± 0.73 m/s. Control group: -Before: 10.00 ± 0.99 m/s. -After: 9.86 ± 0.95 m/s.	Low risk
18	[39] Joris PJ. et al. (2020)	59 overweight or obese adults with (pre)hypertension. Age 60 ± 8 years.	Experimental group: 3.4 g/day of alpha–linolenic acid (ALA). Control group: Sunflower oil. Duration: 12 weeks (3 months).	cfPWV.	SphygmoCor	ALA (vegetable omega-3) may be less potent than EPA/DHA.	No significant effect of ALA on cfPWV (*p* < 0.05). ALA group: -Before: 8.9 ± 1.1 m/s. -After: 8.9 ± 1.1 m/s. Control group: -Before: 8.7 ± 1.1 m/s. -After: 8.8 ± 1.1 m/s.	Some concerns
19	[40] Krantz MJ et al. (2015)	62 patients with hypertension. Age 61.1 ± 10.3 years.	Dosage: 4 capsules daily of 465 mg EPA and 375 mg DHA (3.36 grams). Duration: 3 months.	baPWV.	Does not declare equipment.	Numerical imbalance of subjects between the groups.	No significant effect of omega-3 on baPWV (*p* = 0.36). Omega-3 group: -Before 16.02 ± 0.324 m/s. -After –0.97 m/s. Control group: -Before 16.90 ± 0.335 m/s. -After –0.33 m/s.	Some concerns
20	[41] Kristensen S. et al. (2016)	145 patients with psoriatic arthritis. Age 52.0 ± 11.5 years.	6 capsules with 3 g of omega-3 (50% EPA and 50% DHA) or 3 g of olive oil (control group). For 24 weeks (6 months).	cfPWV or carotid radial (crPWV).	SphygmoCor.	Olive oil is not an inert placebo. They used the cfPWV and crPWV interchangeably.	No significant effect of omega-3 on PWV (*p* = 0.70). Omega-3 group: -Before: 7.66 m/s. -After: 7.61 m/s. Control group: -Before: 7.40 m/s. -After: 7.48 m/s.	Low risk

Values expressed as mean and standard deviation or as median and interquartile range.

Summary of main findings

In this set of 20 randomized controlled trials, supplementation with omega-3 fatty acids (EPA/DHA or ALA) demonstrated heterogeneous effects on arterial stiffness, as measured by pulse wave velocity (PWV). A simple count reveals that slightly more than half of the studies (*n* = 11; 55%) reported statistically significant reductions in at least one PWV index (e.g., carotid–femoral, brachial–ankle, or central PWV), while the remaining nine studies (*n* = 9; 45%) found no significant changes. However, a more analytical synthesis of the data reveals distinct patterns that may help explain this variability. Specifically, the likelihood of observing a significant reduction in PWV appeared to be associated with three key study characteristics: dosage, intervention duration, and baseline cardiovascular risk of the population.

Favorable effects were more frequently observed in studies using moderate to high doses of EPA/DHA (approximately 1.8 to 4 g/day) [23,24,25,26,27,28,29,30,31] and interventions lasting 12 weeks or longer [23,24,25,26,27,28,29,30,31,32,33]. Furthermore, significant improvements were more consistently reported in populations with preexisting metabolic disorders or elevated vascular risk, including individuals with metabolic syndrome [34], dyslipidemia [23,24], diabetes with neuropathy [33], abdominal aortic aneurysm [30], and older adults [32]. In contrast, studies that reported null results tended to feature lower doses of omega-3s [34,35,36,37,38], acute or short-term interventions [36,38], or the use of ALA (a plant-based omega-3) instead of EPA/DHA [34,39]. However, most studies used EPA/DHA, had interventions longer than 12 weeks, large samples, and low risk-of-bias ratings on the RoB 2 tool. Still, some showed significant effects of omega-3 on PWV while others did not.

It is important to emphasize that these patterns represent observational trends derived from comparing study designs and outcomes, rather than definitive causal relationships. The observed associations between dose, duration, population, and effect on PWV should be interpreted with caution, as they may be confounded by other variables such as differences in baseline PWV values, adherence rates, concurrent medications, and the specific PWV measurement site used. Table 2 provides a structured overview of the number of studies with and without significant effects on PWV, stratified by dose type of omega-3, intervention time, study size and quality, to further illustrate these trends.

## 4. Discussion

The purpose of this systematic review was to examine the effect of omega-3 polyunsaturated fatty acid supplementation on vascular stiffness, measured using pulse wave velocity (PWV), across 20 studies. The analysis considered both supplements and dietary sources of omega-3 in relation to PWV. Although the majority of reviewed studies reported a reduction in PWV, a considerable number observed no statistically significant effects. These inconsistent findings likely reflect clinical heterogeneity among patient populations, as well as differences in the form and duration of omega-3 administration. Such variability underscores the need for more standardized protocols and larger, well-controlled trials to clarify the conditions under which omega-3 supplementation may effectively improve vascular stiffness.

### 4.1. Clinical–Methodological Heterogeneity and Its Impact on Results

Considerable heterogeneity was observed across studies regarding population characteristics (age, comorbidities, cardiovascular risk), omega-3 dose and type (EPA/DHA vs. ALA), intervention duration, use of non-inert placebos (e.g., olive oil), and measurement methodology, complicating direct comparisons and quantitative synthesis. Age, sex, systolic blood pressure (SBP), and heart rate have been identified as determinants of PWV [36]. In one study, older populations exhibited higher PWV than younger individuals. Daily supplementation with EPA and DHA for 12 weeks was associated with a reduction in carotid–femoral PWV (cfPWV) in older participants, while no such reduction was observed in younger individuals [32]. This disparity suggests that age may modulate the effect, although the underlying mechanisms remain unclear and require further investigation. The authors suggested that the short duration of the intervention (12 weeks) may limit the extent to which structural vascular changes can be inferred, but their data do not allow for a direct distinction between functional and structural adaptations [32]. It is also possible that baseline vascular health in younger individuals leaves less room for measurable improvement, but this is a hypothesis to be tested. In this regard, of the three studies that included young subjects under 30 years of age [25,32,35], significant effects of omega-3 supplementation were observed only in smokers [25]. This finding supports the hypothesis that vascular benefit appears to depend on the presence of preexisting vascular damage, such as the accelerated arterial aging induced by smoking, which confers upon these subjects a vascular age greater than would be expected for their chronological age.

Men over 30 years exhibit higher PWV than women of the same age, possibly due to estrogen’s protective effects [30]. A positive correlation exists between SBP and PWV, with each 1 mmHg increase in SBP corresponding to a 0.073 m/s rise in PWV [36]. However, cfPWV reductions in older adults supplemented with omega-3 occurred independently of blood pressure or arterial wave reflection effects [32]. Another study reported that omega-3 supplementation significantly reduced heart rate after 12 weeks, suggesting a possible indirect effect on PWV mediated by heart rate modulation [30].

Regarding comorbidities, Casanova et al. [28] observed that, in a subgroup of hypertensive patients with hypertriglyceridemia and high cardiovascular risk, significant PWV reductions were found after omega-3 administration. Similarly, in patients with metabolic syndrome [26] and in those with abdominal aortic aneurysm [30], PWV showed linear decreases following omega-3 treatment. In type 2 diabetes patients, omega-3 supplementation was associated with a reduction in PWV and aortic augmentation index [33]; however, as with the previous studies, these findings should be interpreted in light of the particular characteristics of the study population, thereby limiting their generalizability to other clinical groups. Another study found no significant PWV changes in dyslipidemic patients with coronary artery disease receiving statin monotherapy versus combined statin-EPA therapy; however, after multivariate adjustment for age and blood pressure, regression analysis suggested an association between PWV reduction and combined treatment [24]. In contrast, hypertensive patients with hypertriglyceridemia but low cardiovascular risk showed no PWV changes after omega-3 administration [28]. McManus, S et al. [36] reported no acute (4 h postprandial) effects of DHA- or EPA-rich diets on PWV in a group of healthy men aged 36–54. Patients with non-alcoholic fatty liver disease exhibited no significant PWV differences between low-fat and Mediterranean diets (higher in monounsaturated fats) [42]. These findings are not conclusive because they were derived from small and specific populations. Recently, it has been reported that the intake of polyunsaturated fatty acids and omega-3 fatty acids through fish consumption could be associated with lower peripheral arterial stiffness [43]. Furthermore, the profile of individuals at higher risk of elevated vascular age and cardiac age includes older men who are characterized by low socioeconomic status, a sedentary lifestyle, regular alcohol consumption, and poor adherence to the Mediterranean diet [44]. The strongest evidence appears to indicate that omega-3 has a long-term significant effect in reducing arterial stiffness as measured by pulse wave velocity. However, the preexisting state of the vascular bed, endothelial function, and other factors such as liver function and gut microbiota status appear to influence the efficacy of omega-3 in reducing arterial stiffness. All of these observations are qualitative and based on comparisons across studies, so they should be interpreted with caution.

### 4.2. Biological Plausibility and Concordance with Proposed Mechanisms

Omega-3 fatty acids have plausible mechanisms for improving vascular function: anti-inflammatory effects [26,32], increased nitric oxide bioavailability [26,28], reduction in triglycerides [32], and changes in membrane phospholipid composition [28], which affect vasomotor responses. At the experimental level, it has been shown that omega-3 accumulates at the sn-2 position of membrane phospholipids, competing with arachidonic acid for enzymes involved in the synthesis of pro-inflammatory mediators, thereby generating eicosanoids with lower pro-inflammatory, platelet-aggregating, and vasoconstrictor activity [34]. Clinical evidence indicates that omega-3 supplementation reduces serum levels of C-reactive protein [32] and interleukin-6 [27], which has been associated with decreased arterial stiffness. Likewise, endothelial function improves, reflected in an increase in flow-mediated dilation (FMD) in supplemented patients [27,33], which may be due to anti-inflammatory effects or modulation of oxidative stress, although the exact mechanism has not been fully elucidated [24].

**Figure 2:**
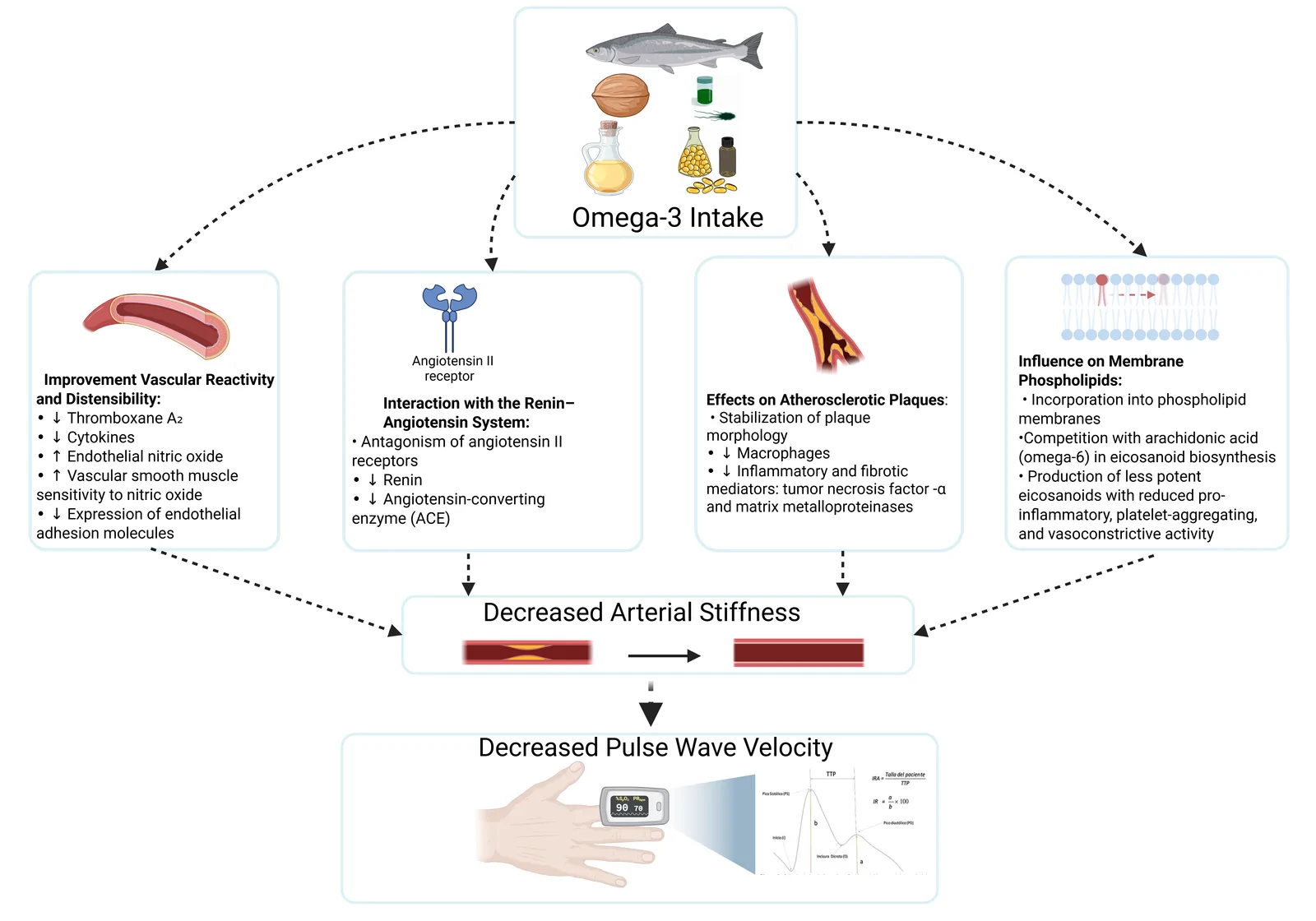
Physiological mechanisms by which omega-3 intake affects arterial stiffness. Source: Author’s own work.↑ Increase ↓Decrease.

Figure 2 shows the possible mechanisms by which omega-3 improves arterial stiffness. These mechanisms are consistent with functional effects in peripheral vessels or in early stages of dysfunction. However, central arterial stiffness reflects structural changes in the media (elastin/collagen) that may require longer periods or combined interventions to show reversibility; therefore, functional improvements do not always translate into sustained reductions in cfPWV, particularly in populations with advanced vascular disease.

### 4.3. Influence of Dose, Duration, and Composition

There is a trend for doses ≥1.8 g/day of EPA/DHA and treatments lasting at least 12 weeks to be more likely to produce significant reductions in PWV. Studies employing high doses show variable results [38,40], suggesting that dose alone does not guarantee an effect and that intervention duration, population characteristics, and baseline vascular status are important modulators. It has been proposed that DHA exerts a greater beneficial effect than EPA [36]. Moreover, studies with ALA generally did not demonstrate a clear benefit on PWV [30,33,38], indicating lower potency of ALA than EPA/DHA in modifying arterial stiffness within the studied timeframe. Conversely, the use of active placebos (olive oil) [30,38,41] may attenuate differences between groups if they exert their own vascular effects, biasing results toward the null.

In summary, the efficacy of omega-3 supplementation in reducing arterial stiffness may be modulated by dose, duration, and composition: higher doses (≥1.8 g/day) and prolonged treatment (≥12 weeks) increase the likelihood of benefit, although individual factors such as baseline vascular status also play a role. DHA appears to be more potent than EPA, whereas ALA shows little effect over the typical timeframes of available studies. Furthermore, the choice of placebo (e.g., olive oil) may mask the true treatment effects. Importantly, these observations remain qualitative and derive from cross-study comparisons rather than direct head-to-head trials, and they should therefore be interpreted as trends rather than conclusive evidence.

### 4.4. Interpretation by Arterial Segment and Measurement Methodology

Results differ according to the evaluated arterial segment and the device used. Crucially, carotid–femoral pulse wave velocity (cfPWV) and brachial–ankle pulse wave velocity (baPWV) are not interchangeable because they assess different arterial territories: cfPWV reflects central (aortic) stiffness, whereas baPWV integrates central and peripheral (muscular) arterial segments. This distinction has direct clinical implications. Carotid–femoral PWV, considered the gold standard for assessing central arterial stiffness, yielded mixed results regarding the effects of omega-3, with several studies showing no significant effects [18,28,32,34,36,38,39,41] and othersreporting significant reductions [25,26,28,30,32]. Some trials report significant benefits in subgroups (e.g., patients with metabolic syndrome [26], abdominal aortic aneurysm [30], smokers [25], high cardiovascular risk [28] and older adults [32]), but large and/or well-controlled studies in general populations often found no effect [18,32,34,38,41]. Clinically, a lack of change in cfPWV suggests that omega-3 may not induce structural remodeling of the aorta in unselected populations, whereas positive findings in high-risk subgroups could indicate a potential role in targeted prevention.

In contrast, baPWV measured by oscillometric or plethysmographic methods shows a near–equal number of studies reporting absence of effect [24,34,35,40] and favorable changes [23,27,31]. This peripheral measure appears more sensitive to post-intervention changes, possibly reflecting greater functional plasticity of peripheral vessels or higher sensitivity of oscillometric/plethysmographic methods to acute or subacute hemodynamic shifts. However, these methods are more susceptible to variations in blood pressure, blood volume, and local vascular tone, which could lead to detecting transient functional effects rather than durable structural alterations. From a clinical standpoint, improvements in baPWV should be interpreted cautiously: they may signal early or transient vascular benefits, but cannot be directly extrapolated to central arterial stiffness. Therefore, cfPWV and baPWV should be used as complementary, not interchangeable, indices; cfPWV remains the preferred surrogate for cardiovascular risk prediction, while baPWV may serve as a more accessible screening tool for detecting acute changes in vascular function.

### 4.5. Bias, Quality, and Statistical Considerations

Several trials included in this review present limitations that increase the risk of bias and reduce the certainty of the evidence. These limitations can be categorized by the type of bias they introduce:

• Performance and detection bias: Many studies lacked a double-blind design, which can lead to biased intervention delivery and outcome assessment. Additionally, most trials did not control for dietary omega-3 intake, allowing variations in background intake that may confound the effects of supplementation.
• Confounding bias: Heterogeneity in concomitant treatments (e.g., statins, fibrates) across studies further complicates interpretation, as these medications can independently affect arterial stiffness.
• Multiplicity (reporting) bias: Some trials performed subgroup analyses without adjusting for multiple comparisons, increasing the risk of false-positive findings.
• Imprecision and low statistical power: Small sample sizes and short study durations [35,36,38] limit the reliability of estimates and produce inconsistent results, raising the likelihood that reported effects may be inflated or spurious.

Together, these issues suggest that positive findings may be restricted to specific populations or be methodological artifacts, as indicated by larger, better-controlled studies that found no effect [17]. Nevertheless, the strength of this review lies in summarizing studies that demonstrate a beneficial effect of omega-3 supplementation on arterial stiffness as measured by PWV. It shows that this effect is not universal but depends on several factors, including the patient’s clinical conditions and the form and timing of omega-3 administration. The principal mechanisms implicated in the effects of omega-3 on arterial stiffness are improvements in endothelial function and anti-inflammatory actions, although many others may be involved.

Additionally, the specificity of the search strategy employed should be acknowledged as a limitation. Although a precise syntax (“omega-3” AND “pulse wave velocity”) was chosen to ensure the relevance of the retrieved records and the feasibility of the review process across three major databases (Scopus, PubMed, and Web of Science), this approach restricts search sensitivity. It is possible that not all potentially relevant studies were identified, particularly those using alternative terminology for either the intervention or the outcome. With respect to the intervention, studies may refer to omega-3 fatty acids using broader terms such as “n-3 fatty acids”, “fish oil”, “eicosapentaenoic acid (EPA)”, “docosahexaenoic acid (DHA)”, “alpha-linolenic acid (ALA)” or their combinations, rather than the generic term “omega-3”. Similarly, arterial stiffness may have been reported using related constructs such as “aortic stiffness”, “vascular stiffness”, “arterial distensibility”, or “augmentation index”, without explicitly including the descriptor “pulse wave velocity” (PWV) in the title or abstract, even though the study may have measured PWV as part of its outcomes.

The potential impact of this limitation on the evidence synthesis should be considered with caution. Given that the studies included in this review reported positive and null findings in roughly equal proportions, it is not evident that the omission of studies due to terminological restrictions would introduce a clear directional bias into the conclusions. That is, if the potentially unidentified studies are evenly distributed between findings favorable and unfavorable to omega-3 (a plausible though unverifiable assumption), their absence would not substantially alter the central conclusion of this review: the effect of omega-3 on PWV is variable and dependent on factors such as the population studied, dose, and intervention duration. Nevertheless, the possible loss of studies may have limited the diversity of populations, formulations, or clinical contexts represented, thereby restricting the breadth of evidence available for subgroup analyses. Consequently, this methodological restriction should be weighed when interpreting the comprehensiveness of the topical coverage, but not necessarily the direction of the conclusions reached.

## 5. Conclusions

The findings of this systematic review suggest that the effect of omega-3 fatty acids on pulse wave velocity is variable and appears to depend on multiple interacting factors, including population characteristics, dosage, and intervention duration. Although a beneficial impact was observed more consistently in certain subgroups and in studies employing adequate doses over extended periods, these observations should be interpreted with caution. The marked heterogeneity across studies in terms of design, participant characteristics, and outcome assessment precludes definitive conclusions and underscores the exploratory nature of the trends identified. Rather than providing firm recommendations, the present review highlights the need for further well-designed, standardized trials to clarify the underlying mechanisms and to establish whether specific subgroups may derive greater benefit. Until such evidence becomes available, any clinical recommendations derived from the current body of literature should remain tentative and tailored to individual patient profiles.

## Figures and Tables

**Table 2:** Number of articles with and without significant effects of omega-3 on PWV according to dose, type of omega-3, intervention time, study size and quality.

Effect of Omega-3 (*n* = 20)	Omega-3 Dosage		Type of Omega-3		Intervention Time		Study Size		Quality (RoB 2 Scale)		
	High (1.8–4 g/d)	Low (<1.8 g/d)	ALA	EPA/ DHA	Chronic (≥12 Weeks)	Acute (Single Dose)	Small (*n* < 30)	Large (*n* ≥ 30)	High Risk	Low Risk	Some Concerns
With significant effect (*n* = 11)	9	2	1	10	11	0	4	7	4	4	3
No significant effect (*n* = 9)	4	5	2	7	7	2	2	7	0	5	4

## Data Availability

No new data were created or analyzed in this study.

## Supplementary Materials

The following supporting information can be downloaded at https://www.mdpi.com/article/10.3390/medicina62081553/s1, Table S1: PRISMA 2020 checklist.

## Abbreviations

The following abbreviations are used in this manuscript:

AA	Arachidonic acid
AAA	Abdominal aortic aneurysm
baPWV	Brachial–ankle pulse wave velocity
cfPWV	Carotid–femoral pulse wave velocity
cPWV	Central pulse wave velocity
crPWV	Carotid radial pulse wave velocity
DHA	Docosahexaenoic acid
EPA	Eicosapentaenoic acid
FMD	Flow-mediated dilation
LA	Linoleic acid
PWV	Pulse wave velocity
SBP	Systolic blood pressure
